# Obesity-induced cardiac lipid accumulation in adult mice is modulated by G protein-coupled receptor kinase 2 levels

**DOI:** 10.1186/s12933-016-0474-6

**Published:** 2016-11-10

**Authors:** Elisa Lucas, Rocio Vila-Bedmar, Alba C. Arcones, Marta Cruces-Sande, Victoria Cachofeiro, Federico Mayor, Cristina Murga

**Affiliations:** 1 Departamento de Biología Molecular and Centro de Biología Molecular Severo Ochoa (UAM-CSIC), C/Nicolas Cabrera 1, 28049 Madrid, Spain; 2 Instituto de Investigación Sanitaria La Princesa, Madrid, Spain; 3 Departamento de Fisiología, Facultad de Medicina, Universidad Complutense, Madrid, Spain; 4 Instituto de Investigación Sanitaria Gregorio Marañón (IiSGM), Madrid, Spain

**Keywords:** Cardiac steatosis, Obesity, Insulin resistance, G protein-coupled receptor kinase 2, Cardiac hypertrophy, Mitochondria

## Abstract

**Background:**

The leading cause of death among the obese population is heart failure and stroke prompted by structural and functional changes in the heart. The molecular mechanisms that underlie obesity-related cardiac remodeling are complex, and include hemodynamic and metabolic alterations that ultimately affect the functionality of the myocardium. G protein-coupled receptor kinase 2 (GRK2) is an ubiquitous kinase able to desensitize the active form of several G protein-coupled receptors (GPCR) and is known to play an important role in cardiac GPCR modulation. GRK2 has also been recently identified as a negative modulator of insulin signaling and systemic insulin resistance.

**Methods:**

We investigated the effects elicited by GRK2 downregulation in obesity-related cardiac remodeling. For this aim, we used  9 month-old wild type (WT) and GRK2+/− mice, which display circa 50% lower levels of this kinase, fed with either a standard or a high fat diet (HFD) for 30 weeks. In these mice we studied different parameters related to cardiac growth and lipid accumulation.

**Results:**

We find that GRK2+/− mice are protected from obesity-promoted cardiac and cardiomyocyte hypertrophy and fibrosis. Moreover, the marked intracellular lipid accumulation caused by a HFD in the heart is not observed in these mice. Interestingly, HFD significantly increases cardiac GRK2 levels in WT but not in GRK2+/− mice, suggesting that the beneficial phenotype observed in hemizygous animals correlates with the maintenance of GRK2 levels below a pathological threshold. Low GRK2 protein levels are able to keep the PKA/CREB pathway active and to prevent HFD-induced downregulation of key fatty acid metabolism modulators such as Peroxisome proliferator-activated receptor gamma co-activators (PGC1), thus preserving the expression of cardioprotective proteins such as mitochondrial fusion markers mitofusin MFN1 and OPA1.

**Conclusions:**

Our data further define the cellular processes and molecular mechanisms by which GRK2 down-regulation is cardioprotective during diet-induced obesity, reinforcing the protective effect of maintaining low levels of GRK2 under nutritional stress, and showing a role for this kinase in obesity-induced cardiac remodeling and steatosis.

**Electronic supplementary material:**

The online version of this article (doi:10.1186/s12933-016-0474-6) contains supplementary material, which is available to authorized users.

## Background

Obesity is a complex condition that affects virtually all age and socioeconomic groups and threatens to overwhelm both developed and developing countries. The growing incidence of obesity is particularly preoccupying given its strong association with cardiovascular disease and overall mortality. Although obesity is most commonly caused by a disruption in energy homeostasis due to the imbalance between dietary energy consumption (calorie-dense food and drinks) relative to energy expenditure (energy loss via metabolic and physical activity), the etiology of obesity is highly complex and includes several factors that promote an increase in body fat mass [1].

Besides an altered metabolic profile, a variety of adaptations/alterations in cardiac structure and function occur in the individual as adipose tissue and lipids accumulate in excessive amounts, even in the absence of comorbidities such as type 2 diabetes or hypertension [2]. For instance, the mass of the left ventricle has been shown to grow and correlate proportionally with body weight [3]. Eventually, prolonged persistence of obesity causes both left ventricular systolic and diastolic dysfunctions [4]. In humans, increased cardiac mass has been postulated to result from epicardial fat deposition and fatty infiltration of the myocardium [5]. In fact, triglyceride content in human cardiac tissue is increased in obese compared with normal-weight subjects [6]. Accumulation of intra-myocellular triglycerides in the heart is also a commonly described feature of most animal models of obesity [7, 8]. The ectopic presence of triglycerides and lipid metabolites such as ceramides has been related to lipotoxicity and cardiomyocyte apoptosis [9]. Interestingly, a palmitic acid-ceramide pathway accounts for impaired insulin sensitivity [10], whereas ceramide inhibition has been suggested to be an effective deterrent to heart disease risk in conditions like hyperinsulinemia [11]. In fact, a positive correlation between cardiac lipid accumulation and cardiac dysfunction has been established giving rise to the term lipotoxic cardiomyopathy.

Another common feature of the obese heart is impaired insulin signaling. It starts to develop within 2 weeks of high fat diet (HFD) in animal models, and represents an early adaptation of the heart to caloric excess that promotes the development of diabetic cardiomyopathy [12, 13]. Interestingly, intra-myocellular lipid content appears to better predict muscle insulin resistance than fat mass in lean individuals and non-obese, non-diabetic but insulin-resistant adults and children (see references in [14]). This condition not only alters cardiac metabolism, but also increases myocardial oxygen consumption, reduces cardiac efficiency by uncoupling of the mitochondria and increases oxidative stress [15].

G protein-coupled receptor kinase 2 (GRK2) is a serine/threonine kinase originally discovered to regulate G protein-coupled receptor (GPCR) desensitization and known to play an important role in cardiac function and dysfunction [16, 17]. GRK2 expression increases in different cardiac hypertrophy and heart failure human conditions [16, 17]. Interestingly, GRK2 is emerging as an important signaling hub with a complex interactome and has recently been identified as a direct modulator of insulin signaling in several tissues, including the heart [18, 19]. Interestingly, GRK2+/− mice (expressing some 50% less protein than control littermates) show improved systemic insulin sensitivity in different insulin resistance models [19, 20], and accordingly, inducible GRK2 downmodulation reverts key features associated to the diabetic phenotype in HFD-fed mice [21]. On the other hand, we have recently described that GRK2 levels are increased in the hearts of adult ob/ob mice as well as in mice fed with a HFD for 12 weeks [19].

Given the emerging role of GRK2 as a regulatory hub in heart metabolism and physiology, we have explored the role of GRK2 dosage in the development of obesity-induced cardiac remodeling and steatosis in 9 month-old mice, since obesity-related cardiac pathological events become more prevalent during adulthood. Our results show that decreased GRK2 protein levels is per se able to prevent intra-myocellular lipid accumulation, cardiac steatosis, fibrosis and hypertrophy promoted by the long-term HFD feeding, by mechanisms involving increased expression of markers of mitochondrial fusion (such as MFN1 and L-OPA1/S-OPA1 ratio) and fatty acid oxidation regulation (such as PGC1) downstream of the PKA/CREB cascade.

## Methods

### Animals

Experiments were performed on male wild type (WT) and hemizygous-GRK2 (GRK2+/−) mice maintained on the C57BL/6 background. The animals were bred and housed on a 12-h light/dark cycle with free access to food and water. GRK2+/− mice and their corresponding WT littermates were fed ad libitum either an standard diet (SD, providing 13% of total calories as fat, 67% as carbohydrate and 20% as protein; 2014S Rodent Maintenance Diet, Teklad, Harlan, Barcelona, Spain) or a high fat diet (HFD, providing 45% of total calories as fat, 35% as carbohydrate and 20% as protein, Rodent Diet D12451, Research Diets, New Brunswick, NJ, USA) for 30 weeks. Animals were maintained at a room temperature of 22 ± 2 °C with a relative humidity of 50 ± 10% and under pathogen-free conditions. Body weight and food intake were measured weekly.

### Metabolic assays

Insulin tolerance tests (ITT) were performed as previously described [22]. Animals were fasted for 4 h, and baseline blood samples were collected from the tail. Insulin (0.8 U/kg body weight) was administered by i.p. injection, and blood samples were taken 15, 30 and 60 min after injection. Glucose concentration (mg/dl) was determined using an automatic analyzer (One Touch Ultra), from Life Scan.

### Heart collection and processing

Mice were euthanized using CO_2_ and weighted. Hearts were surgically removed, washed, dried and immediately weighted. Auricles were removed and ventricles were sliced transversally in four portions. The two central slices were fixed in 4% paraformaldehyde and embedded in paraffin or Tissue-Tek^®^ OCT for histological analysis. The other two portions were frozen in liquid nitrogen for protein and gene expression analysis.

### Cardiomyocyte hypertrophy determination

Paraffin blocks of heart slices were cut in 6 μm-thick slices and stained with Masson’s trichrome for the evaluation of cardiomyocyte area. Digital images of transversally cut cardiomyocytes were captured using a light microscope (Olympus, Germany) at 20× magnification. Four mice were employed for each condition (three fields per heart), and cardiomyocyte size was calculated by quantitation of 150–200 cells per field using image analysis software (ImageJ).

### Fibrosis staining and quantitation

Fibrosis was quantified in Picro-sirius red-stained sections in order to detect collagen fibers. The area of interstitial fibrosis was identified, after excluding the vessel area from the region of interest, as the ratio of interstitial fibrosis or collagen deposition to total tissue area and expressed as %CVF (collagen volume fraction). For each heart, 10–15 fields were analyzed with a 40× objective lens under transmitted light microscopy (Leica DM 2000; Leica AG, Germany). All measurements were performed blind in an automated image analysis system (Leica LAS,4.3; Leica AG, Germany). Images were calibrated with known standards. A single researcher unaware of the experimental groups performed the analysis.

### Gene expression analysis

mRNA from heart tissue of at least six mice per condition was isolated as described in [19]. RT-PCRs were performed by the Genomic Facility at Centro de Biologia Molecular “Severo Ochoa” (CBMSO, Madrid), using Light Cycler equipment (Roche, Indianapolis, IN, USA). Gene expression quantifications were performed using both commercial Taqman Gene Expression Assay probes (Applied Biosystems, Life Technologies, Grand Island, NY, USA) and self-designed probes purchased from Sigma labeled with Syber Green (see Additional file 1: Table S1). qPCRs and statistical analysis of the data were performed by the Genomic Facility using GenEx software. A geometric mean of two stably expressed and commonly used reference genes (hprt and rps29) was used for data normalization.

### Western Blot analysis

Heart tissue was homogenized as described in [19]. Typically 40 μg of total cardiac protein was resolved per lane by SDS-PAGE and transferred to a nitrocellulose membrane. Blots were probed with specific antibodies against GRK2 (sc-562), PKA (sc-903), GAPDH (sc-32233) and nucleolin (sc-13057) from Santa Cruz Biotechnology, Dallas, TX, USA; CREB (9198), P-CREB (Ser 133) (9198), AMPK (2532), P-AMPK (Thr 172) (2535) and P-PKA (Thr 197) (4781) from Cell Signalling Technology, Danvers, MA, USA; MFN1 (ab57602) from Abcam, Cambridge, UK; and OPA1 (612606) from BD Transduction Laboratories San Jose, CA, USA.

### Intracellular lipid droplet quantification

OCT frozen blocks of heart tissue were cut in 6 μm-thick slices, mounted on 10% glycerol in PBS-DAPI (5 ng/μl) to visualize the nucleus, and stained with Oil red O as described [23]. All sections were examined using a fluorescence resonance energy transfer (FRET) equipment coupled to an inverted Axiovert200 (Zeiss, Germany) microscope in the Confocal Microscopy Facility of our center. Oil red O-stained sections were examined in epifluorescence using a DsRed (500–650 nm) and DAPI (359–371 nm) excitation filter. Digital images of arbitrary fields were captured at 100× magnification from three different hearts (ten fields per mouse). Total lipid droplet content per total cell area and droplet areas within each field were determined using image analysis software (ImageJ).

### Statistical analysis

All data are expressed as mean values ±SEM and N represents the number of animals. Statistical significance was analyzed using unpaired two-tail Student’s t test except when repeated measures were taken over time in the same group of animals when a two-way ANOVA followed by Bonferroni’s post hoc test was used. All data were analyzed using GraphPad Prism software. Differences were considered statistically significant when P < 0.05.

## Results

### Decreased levels of GRK2 attenuates the diet-induced obesity phenotype

**Fig. 1:**
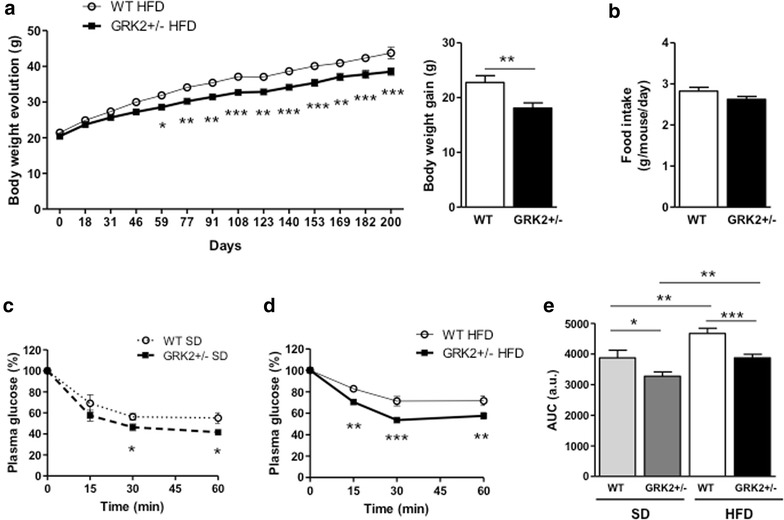
GRK2+/− animals show an attenuated obese and insulin-resistant phenotype after long-term HFD feeding. **a** Comparison of body weight evolution and body weight gain (the former analyzed by two-way ANOVA followed by Bonferroni post hoc test) between WT and GRK2+/− animals after 30 weeks of HFD feeding. **b** Daily food intake (statistical analysis: two-tailed unpaired T test). **c**, **d** Intraperitoneal insulin tolerance tests (ITT) in SD- and HFD-fed mice (statistical analysis: two-way ANOVA), and **e** histogram showing the product of ITTs area under the curve (AUC). Data in all *panels* are mean ± SEM with N = 6–7 per genotype and condition using unpaired two-tail Student’s t test analysis except where indicated. *P < 0.05, **P < 0.01, ***P < 0.001

Two months after birth mice were fed either a SD or HFD for 30 weeks. While both genotypes significantly gained weight after HFD feeding compared with SD-fed mice (see Additional file 1: Figure S1), GRK2+/− mice maintained a significantly leaner phenotype compared with WT animals (Fig. 1a; Additional file 1: Figure S1), in the absence of differences in food intake (Fig. 1b). Since systemic insulin resistance is a common comorbidity in obese individuals, we performed insulin tolerance tests (ITTs) on animals of each genotype. We observed that 9-month old GRK2+/− mice were more sensitive to insulin than their WT littermates in SD conditions (Fig. 1c), in line with previously published results [20]. Interestingly, such enhanced insulin sensitivity detected upon GRK2 downregulation was even more pronounced after a long-term HFD feeding (Fig. 1d) as reflected by analysis of the area under the curve (Fig. 1e). These results build on previous data from our laboratory that showed improved maintenance of body weight and insulin sensitivity in GRK2+/− mice after a 12-week HFD, indicating that these animals are able to preserve these features even after 30 weeks of HFD feeding when they are already 9 months old.

### Lower levels of GRK2 protect hearts from high fat diet-induced hypertrophy and fibrosis

**Fig. 2:**
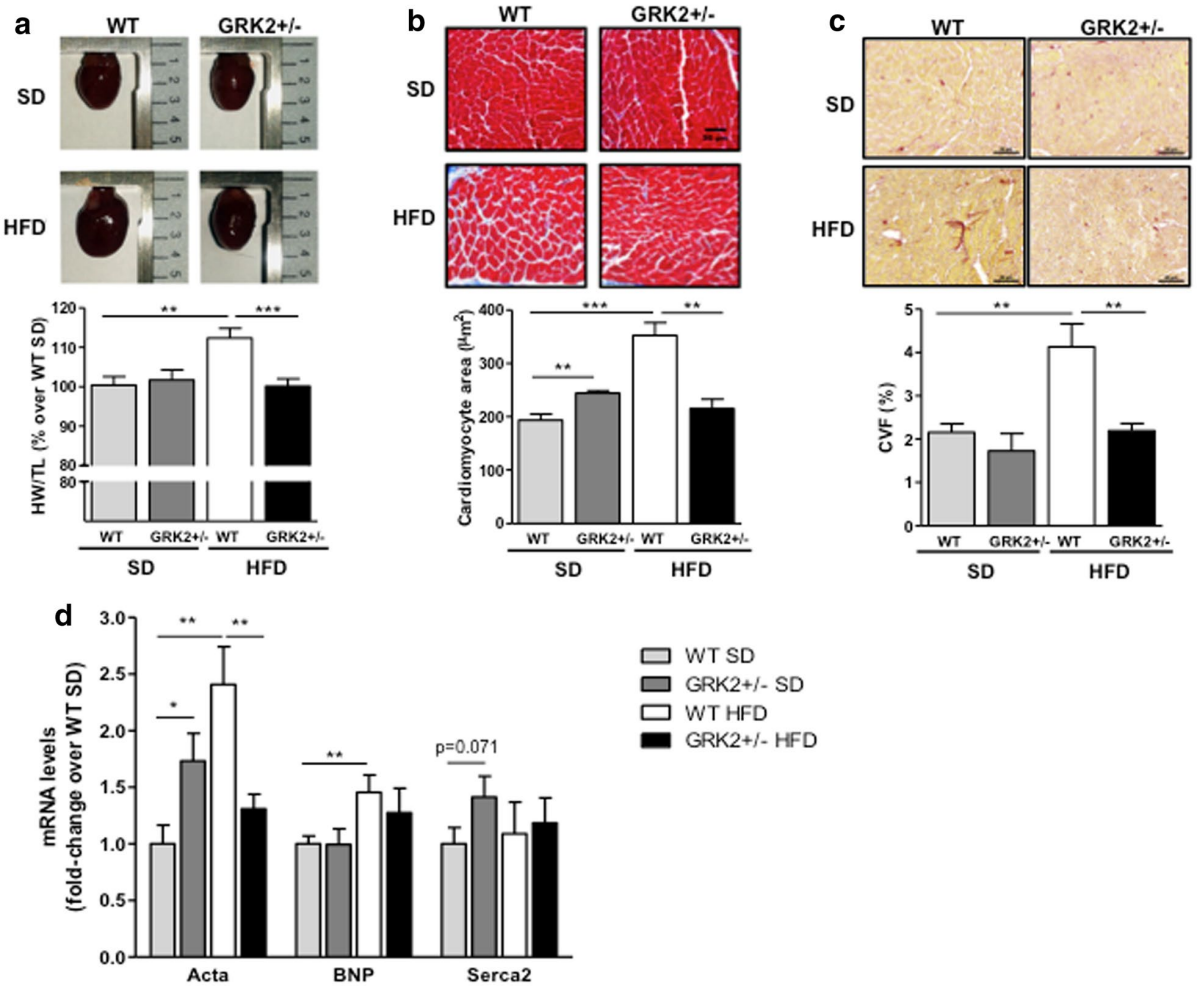
Low levels of GRK2 protect mice from long-term (30 weeks) HFD-induced heart hypertrophy and fibrosis. **a** Heart weight to tibial length ratio in HFD-fed 9 month-old WT and GRK2+/− mice, compared with their littermates fed with standard diet (N = 6–9 per genotype and condition). **b** Cardiomyocyte cross-sectional area from each genotype expressed in μm^2^ (N = 4). **c** The ratio of interstitial fibrosis or collagen deposition to the total tissue area was measured in Picro-sirius red-stained sections and expressed as %CVF (collagen volume fraction) (N = 4–7 animals per genotype and condition). Results are from 10 to 15 photomicrographs from each heart (magnification ×40). **d** Expression of markers of cardiac hypertrophy (Acta1), cardiac stress (BNP) and cardiac functionality (Serca2) were quantified by qPCR and normalized by a geometrical mean of HPRT and RPS29 (N = 5–6). Data are mean ± SEM with a two-tail unpaired Student’s t test statistical analysis.*P < 0.01, ***P < 0.001

In order to determine if obesity-induced cardiac remodeling could be affected by GRK2 dosage we set out to study the hearts of mice fed for 30 weeks with HFD, since 12 weeks were not enough to induce cardiac hypertrophy (data not shown). We first observed that HFD feeding provoked cardiomegaly in WT animals while, in GRK2+/− mice, heart dimensions were indistinguishable from those of SD-fed mice (Fig. 2a). Interestingly, a similar effect was also observed at a cellular level where a reduction of GRK2 prevented the increase in cell size that was induced by HFD in WT mice (Fig. 2b), even considering that hemizygous animals display a mild, non-pathological cardiomyocyte hypertrophy in SD conditions (in agreement with previous reports [19]). The degree of fibrosis as measured by Picro-sirus red staining after 30 weeks of HFD feeding in WT mice doubled that found in SD-fed control animals, while this pathological increase in fibrosis was also absent in HFD-fed GRK2+/− mice (Fig. 2c).

In order to further characterize the cardiac hypertrophy that we detected, we quantified the mRNA expression levels (Fig. 2d) of α-skeletal actin (Acta1), a prototypical marker of cardiac hypertrophy, B-type natriuretic peptide (BNP), a highly sensitive marker of cardiac pathology/stress, and sarco/endoplasmic reticulum Ca^2+^ ATPase (Serca2), a marker of cardiac functionality that is decreased in most cases of hypertrophied failing hearts. The gene expression profile for Acta1 precisely correlates with the degree of cardiomyocyte hypertrophy observed. GRK2+/− mice did not show the pathological increase in BNP expression observed in WT littermates after the HFD. We found no significant changes in Serca2 expression in either genotype or feeding regime, but there is a tendency towards an increased expression of Serca2 in SD-fed GRK2+/− mice compared to SD-fed WT animals. This would indicate a possible amelioration of cardiac functionality upon GRK2 downregulation in this model, in line with previously reported results in other models of cardiac hypertrophy [19, 24]. In sum, we can conclude that low levels of GRK2 prevent the development of obesity-induced cardiomyocyte and heart pathological hypertrophy, and also of cardiac fibrosis after a long-term HFD feeding.

### GRK2+/− mice are protected from HFD-promoted intramyocardial lipid accumulation

**Fig. 3:**
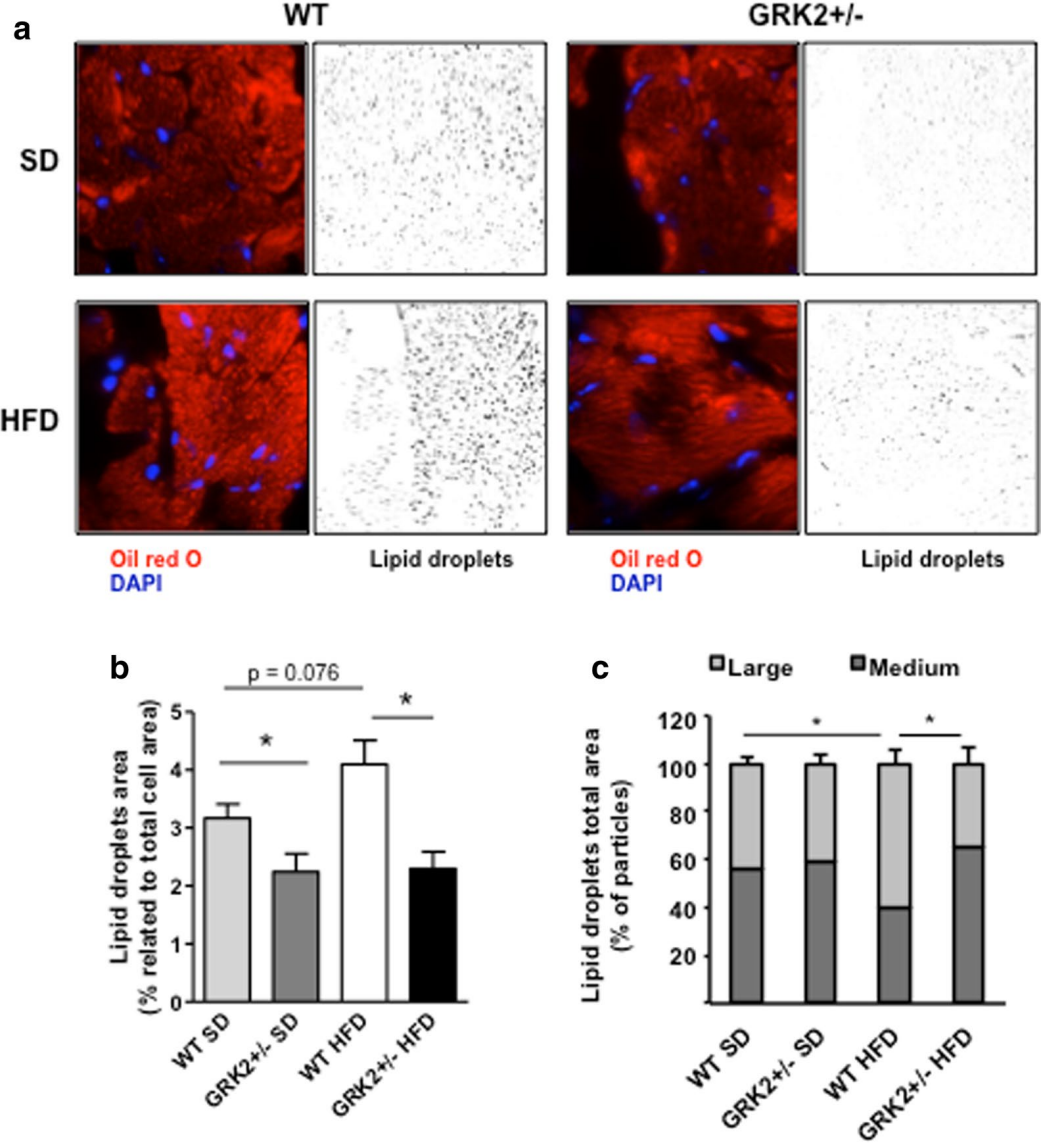
Intracellular lipid accumulation in the heart is enhanced in WT compared with GRK2+/− littermates after standard or long-term HFD feeding. **a** Frozen heart sections were stained with Oil red O and DAPI to visualize and quantify intracellular lipid droplets and nuclei, respectively. Lipid droplets in each condition are shown in *black* and *white* after image processing. Results are from at least ten photomicrographs from each heart (magnification ×100). **b** Percentage of the area occupied by lipid droplets in cardiomyocytes for each condition. **c** Lipid droplets areas were classified according to their sizes as medium (0.5–1 μm^2^) or large (>1 μm^2^). The total area of lipid droplets of each size was normalized with the total area of lipid droplets. Statistical analysis was performed using unpaired two-tail Student’s t test. Data are mean ± SEM (N = 3). *P < 0.05, **P < 0.01

One of the characteristic pathological features of hearts from obese individuals is the accumulation of lipid droplets in the cardiac cell that can promote lipotoxicity. Staining of fat depots with Oil red O (Fig. 3a) showed that the amount of intracellular lipid droplets was significantly lower in either HFD- or SD-fed GRK2+/− mice hearts than in their littermate WT counterparts. The amount of intracellular fat remained unchanged in GRK2+/− mice regardless of the type of diet used, whereas it increased in WT cardiac tissue after HFD feeding (Fig. 3b). Interestingly, the mean size of intracellular lipid depots was significantly larger in cardiomyocytes of HFD-fed WT animals compared to any other condition (Fig. 3c). These differences in cardiac fat depots or in the size of intramyocellular lipids could not be ascribed to changes in the levels of free fatty acids in the plasma of these animals that were not statistically different in GRK2+/− mice compared to WT (see Additional file 1: Figure S2). Of note, the levels of plasma NEFA in WT animals were slightly but significantly reduced after this HFD what does not happen in GRK2+/− mice. This could be ascribed to a deterioration of lipolysis in the WAT of WT mice caused by this long term diet. Consistent with this notion, a preserved lipolytic response of adipocytes to fasting and to β agonists after GRK2 downregulation has been described [21, 25]. Overall, our results suggest that GRK2 downregulation maintains cardiac lipid storage at bay and prevents the pathological accumulation of intramyocellular lipids even in the face of a long-term HFD feeding.

### HFD-dependent increase in GRK2 protein levels correlate with the development of hypertrophy and cardiac steatosis

**Fig. 4:**
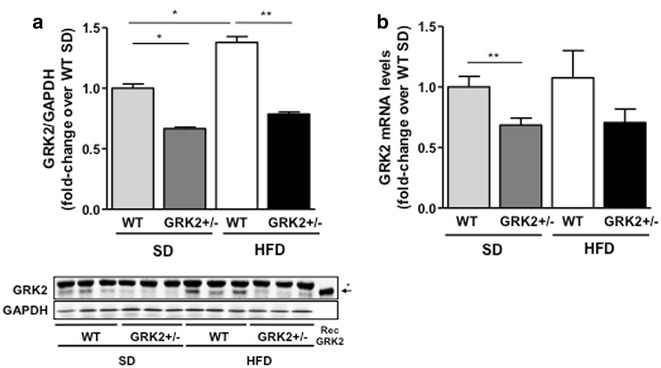
GRK2 protein levels increase in the hearts of long-term HFD-fed animals. **a** GRK2 protein levels in cardiac tissue normalized by GAPDH levels are expressed as fold-increase over SD-fed WT animals (N = 3–4 mice per genotype and condition). A representative blot is shown. The *arrow* indicates the band corresponding to GRK2 (as determined in the *last lane* using 0.5 ng of recombinant purified protein) and the *asterisk* an unspecific band. **b** mRNA levels of GRK2 were quantified by pPCR and normalized by a geometrical mean of HPRT and RPS29 (N = 5–6). Data are mean ± SEM and statistical analysis performed by two-tail unpaired Student’s t test.*P < 0.05, **P < 0.01

In consistency with previous results showing that GRK2 levels were increased upon feeding young animals a HFD for 12 weeks [19], we found that a 30-week long HFD also induced an increase in cardiac GRK2 protein levels, but this increase was only significant in WT mice. GRK2+/− animals maintained significantly lower levels of GRK2 even after the long term HFD feeding (Fig. 4a) that remained comparable to those of SD aged-matched controls. We detected no significant changes in GRK2 mRNA after HFD feeding (Fig. 4b) what suggests that the observed increase in cardiac GRK2 protein levels probably responds to post-translational regulatory mechanisms in agreement with what was previously described [26, 27]. In sum, these data indicate that GRK2 protein levels increase in cardiac tissue in parallel with HFD-induced hypertrophy and steatosis, and that this increase is not detected in GRK2 hemizygous animals.

### GRK2 downregulation keeps the PKA/CREB and AMPK pathways active and promotes higher levels of PGC1 and mitochondrial fusion markers

**Fig. 5:**
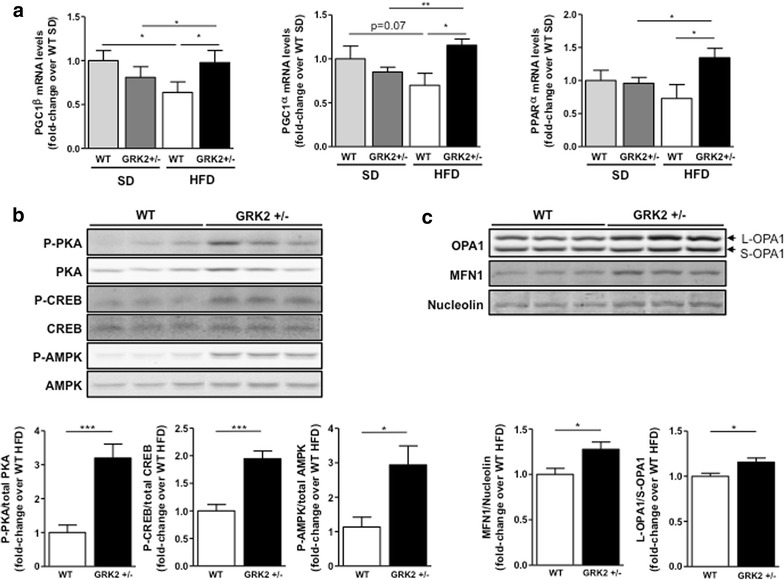
GRK2 downregulation keeps active the PKA/CREB and AMPK pathways and promotes higher levels of PGC1 and mitochondrial fusion markers in cardiac tissue after long-lasting HFD-feeding. **a** Expression of the PPARγ coactivator 1-alpha (ppargc1α), beta (ppargc1β) and of PPARα were quantified by qPCR and normalized by a geometrical mean of HPRT and RPS29 (N = 5–6 animals per genotype and condition). **b** Densitometric analysis and representative blots of total and phospho-PKA (Thr197); total and phospho-CREB (Ser133) and total and phospho-AMPK (Thr172) in WT and GRK2+/− mice after 30 weeks of HFD (N = 5–6). **c** MFN1 and OPA1 protein levels were analyzed in cardiac tissue of the same animals. Graphs display the MFN1 data normalized by Nucleolin levels or the calculated L-OPA1 vs S-OPA1 ratio (N = 5–7). Data are mean ± SEM and are expressed as fold-change over wild type HFD conditions. Statistical analysis was performed by two-tail unpaired Student’s t test. *P < 0.05, **P < 0.01, ***P < 0.001

In order to explore a possible mechanism by which lower GRK2 levels could help prevent intramyocellular lipid accumulation in cardiac tissue, we focused on the family of peroxisome proliferator-activated receptor γ-PPAR γ-coactivator proteins (PGC1). These factors induce the transcription of mitochondrial genes involved in oxidative phosphorylation and fatty acid oxidation, and also of genes governing mitochondrial fusion such as mitofusins (MFN) and optic atrophy 1 (OPA1) [28]. In agreement with what is described for other models of cardiac hypertrophy [28], we observed a tendency towards a decrease in PGC1α/β and in PPARα mRNAs caused by HFD in WT mice. This downregulation is not detected in GRK2+/− hearts where the mRNA levels of these genes are not reduced but instead significantly increased upon HFD feeding (Fig. 5a). No changes in established markers of mitochondrial biogenesis such as TFAM or in mitochondrial-encoded mRNAs such as COI were detected in these samples (Additional file 1: Figure S3). Since PPARα cooperates with PGC1 proteins in the upregulation of genes involved in fatty acid import and β-oxidation in the mitochondria, these data could indicate that a β-oxidative transcriptional profile would remain active in GRK2+/− hearts after a 30-week HFD in the absence of apparent changes in mitochondrial biogenesis.

Several mechanisms are known to promote PGC1 mRNA accumulation. Activation of PKA and consequent phosphorylation of CREB is among the more important ones [28]. A role for up-regulated GRK2 leading to impaired catecholamine responsiveness has been shown in various animal models of cardiac disease including cardiac hypertrophy, whereas inhibiting GRK2 function enhances signaling downstream of beta-adrenergic receptors (βAR) and leads to improved catecholamine sensitivity [24]. Thus, we set out to explore the status of the PKA/CREB cascade in the hearts of these mice. As depicted in Fig. 5b, we can observe a two to threefold increase in the activatory phosphorylation of PKA in Thr197 [29] in GRK2+/− compared to WT HFD-fed mice. This activation of PKA is accompanied by increased phospho-Ser133-CREB, a readout of phosphorylation by PKA that is required for CREB activation [30]. This result shows that the PKA/CREB cascade is more active in GRK2+/− than in WT mice after a long-term HFD and provides an explanation for the increased levels of PGC1α/β mRNA detected in these animals. Besides PKA, other upstream proteins can phosphorylate CREB on Ser133, including AMPK [31]. Moreover, PGC1α is also a known target of AMPK. In this regard, we also observe an increased phosphorylation of AMPK in the hearts of GRK2+/− compared to WT mice after a long-term HFD (Fig. 5b), what may provide an additional mechanism for the increased levels of PGC1α/β mRNA detected in these animals.

Finally, with the aim to determine whether the upregulation of PGC1α/β results in the control of downstream targets, we analyzed the levels of proteins related to mitochondrial fusion known to be induced at least in part by PGC1 transactivation [28]. As shown in Fig. 5c, the protein levels of mitofusin MFN1, a well-established activator of mitochondrial fusion, are significantly larger in the hearts of GRK2+/− animals after a HFD compared to WT mice. Also, the ratio of L-OPA with respect to S-OPA, a known marker of mitochondrial fusion events that correlates with preserved cardiac function [32], is slightly but significantly increased in HFD-fed GRK2+/− mice compared to their WT counterparts. These results may suggest that mitochondrial fusion and, therefore, mitochondrial function could remain more active in the hearts of GRK2 hemizygous mice in the context of a long term HFD feeding.

## Discussion

### Reduced GRK2 protein levels preserve insulin sensitivity after a long-term HFD in adult mice

In this work we present evidence that unmasks a protective role of low GRK2 levels on the effects of obesity-induced cardiac remodeling. We find that adult GRK2+/− mice gain less weight after a long period (30 weeks) of HFD feeding building on our own data describing similar effects after a 12 weeks-long HFD-induced obesity [19, 20], and also when GRK2 was depleted in the middle of the HFD period [21]. Of note, the animals used in this work have reached adulthood (9 months-old at sacrifice), a differential characteristic compared to most studies where rodents initiate the HFD shortly after weaning, and are analyzed when they are still young (typically 4–5 months old). We believe this experimental approach could more reliably mimic the current profile of human obesity whose incidence in middle-age adults is higher than in younger cohorts, as is the presence of associated comorbidities such as cardiovascular disease [33]. Interestingly, insulin sensitivity in GRK2+/− mice is preserved after a HFD compared to WT littermates, indicating that a lower GRK2 dosage is able to protect animals against the development of diet-induced insulin resistance even in adult mice and after long-lasting HFD-induced obesity.

### Obesity-induced pathological cardiac remodeling is prevented by low levels of GRK2

We also report here that GRK2+/− animals appear to be protected from pathological cardiac remodeling induced by obesity. Hemizygous GRK2 mice display neither cardiomegaly nor cardiomyocyte hypertrophy or fibrosis after a HFD of 30 weeks while these alterations are present in control WT mice. Obesity is characterized by an inappropriate expansion and dysfunction of the adipose tissue, and this excessive adiposity provokes structural and functional changes in the heart through hemodynamic (volume overload) and non-hemodynamic factors such as inflammation, hyperglycemia and insulin resistance, altered adipokine secretion, ectopic lipid deposition and lipotoxicity [34]. Since decreased levels of GRK2 attenuate the overall obese phenotype, we cannot discard that the protection against obesity-induced cardiac remodeling observed in GRK2+/− mice could be promoted, at least in part, by the effect of GRK2 downregulation in other tissues. For instance, decreasing levels of GRK2 was shown to improve white adipose tissue lipolysis [21] and increase the thermogenic capacity of brown adipose tissue [25] thus providing effects independent of a specific role of GRK2 in the heart that can affect cardiac steatosis. However, GRK2 downmodulation has been described to be beneficial in other types of cardiac hypertrophy, such as after thoracic aortic constriction, in genetically-based mice models of cardiac dysfunction and after myocardial infarction [35–37], whereas high GRK2 levels correlate well with the degree of heart hypertrophy in different human and murine conditions [16, 37, 38]. So, since the molecular mechanisms underlying cardiomyocyte cell growth in response to different pathological stimuli appear to have common features [39], we believe there are grounds to speculate that cardiac GRK2 could be playing a direct role in modulating obesity-induced heart hypertrophy and cardiac remodeling.

Consistent with this notion, we report an increase in cardiac GRK2 levels in WT HFD-fed mice that correlates with enhanced cardiac hypertrophy and remodeling. Notably, the HFD-induced increase in cardiac GRK2 levels appears to be an early event in the development of obesity-induced cardiac alterations, since it is detected after 12 weeks on HFD in WT animals [19]. At this point insulin resistance is present, not only systemically [20], but also in the heart as determined after acute intravenous insulin injection [19], but no hypertrophy is yet detected in terms of heart weight/tibial length ratio. A similar phenotype has been reported using genetic animal models of obesity (db/db and ob/ob) in which insulin resistance settles at the early onset of the obese phenotype (5–10 weeks after weaning) while left ventricular hypertrophy is only apparent much later [40, 41]. The precise molecular mechanism underlying the HFD-promoted increase in cardiac GRK2 levels remain to be established, although, in the absence of mRNA changes, it is tempting to suggest that insulin resistance may trigger mechanisms modulating protein stability or degradation such as those already described to control the level of this kinase in other tissues and cells [26, 27].

### Low GRK2 helps preserve a cardiac transcriptional profile compatible with lipid catabolism in the face of a long-term HFD

A key feature of HFD-induced cardiac remodeling is lipid accumulation in the myocardium that has been reported to correlate with the degree of obesity [42]. Inappropriate triglyceride deposition into cytoplasmic lipid droplets enlarges the intracellular pool of fatty acyl-CoA thereby providing substrate for oxidative and non-oxidative (e.g. ceramide synthesis [10, 11]) metabolic pathways leading to oxidative stress, cellular dysfunction and apoptosis and insulin resistance [43]. However, such packaging of lipid excess into lipid droplets can also be regarded as an adaptive response of the tissue to accommodate an excessive energy supply while keeping low concentrations of lipotoxic intermediates [44]. We have observed that the amount of lipid droplets is larger in WT than in GRK2+/− mice either after SD or HFD, so GRK2+/− hearts seem to be protected against this ectopic lipid accumulation. In addition, the mean size of intracellular lipid droplets was also smaller in cardiomyocytes of HFD-fed GRK2+/− mice than in WT littermates. Along this line, in skeletal muscle it has been proposed that the reduced lipid droplet size may coincide with increased oxidative enzymatic capacity [45]. Given the already mentioned role of GRK2 in fatty acid handling in white and brown adipose tissues [21, 25], it is tempting to suggest a similar direct effect of GRK2 downregulation in other organs such as the heart, where fatty acid oxidation is the main source of ATP. Notably, GRK2 inhibition has been shown to decrease lipid load in FASN transgenic mice [46]. In fact, both basal respiratory rate (oxygen consumption rate) and ATP-linked respiration, parameters that measure ATP production, were significantly increased when GRK2 was inhibited in mouse cardiomyocytes. Moreover, elevated GRK2 levels negatively regulated myocyte β-oxidation and FA-mediated oxygen consumption when mouse cardiomyocytes were fed with palmitate [47]. Taken together, these data stimulate further research to evaluate whether these novel roles of GRK2 in the regulation of lipid handling in the heart are also relevant in obese patients and if lowering GRK2 is able to preserve cardiac metabolism in the face of dietary lipid excess.

In this regard, the PGC family of coactivators plays a pivotal role in the control of cardiac mitochondrial number and function by regulating transcription of fatty acid oxidation and mitochondrial fusion-related genes. Accordingly, downregulation of PGC1 is a major mechanism in the transition of the oxidative metabolism to a more pathological glycolysis-dependent one observed in hypertrophied hearts and late-stage heart failure [32]. PGC1α is downregulated in numerous rodent models of cardiac hypertrophy or dysfunction (see references in [28]). We also detect a repression of PGC1α/β and of PPARα mRNA after HFD-driven cardiac hypertrophy in WT animals whereas this tendency is not only spared but, rather, inverted in GRK2+/− mice. In our model, PGC1 proteins seem to be primarily acting via binding to PPARα to activate lipid import and catabolism transcription and not via other transcription factors linked to the activation of mitochondrial biogenesis, although this particular conclusion merits further investigation in future studies. In GRK2+/− animals, the maintenance of PGC1α/β mRNA levels could be explained by the elevated activation that we detect of the upstream PKA/CREB route, possibly as a result of the decreased desensitization and enhanced signaling of βAR and other Gs-coupled GPCR that would be expected in the heart of GRK2+/− mice [24]. Interestingly, GRK2 hemizygous animals also show an increased activation of AMPK, an activator of mitochondrial fatty acid oxidation and of PGC-1α expression [45], also suggested to play a protective role in heart failure [48]. Thus, it is tempting to speculate that GRK2 downregulation may enhance AMPK stimulation downstream of several GPCR known to activate cardiac AMPK and to be modulated by GRK2, such as alpha-adrenergic, vasopressin (reviewed in [48]) or adiponectin receptors [46, 49]. An increased activation of AMPK signaling pathway in BAT and WAT of GRK2+/− mice upon cold exposure has also been reported [25].

We also find a preservation of PGC1-dependent MFN1 and OPA1 expression upon long-term HFD feeding in GRK2 hemizygous mice. These mitochondrial fusion markers appear downregulated in animal models of cardiac hypertrophy and in cardiac tissue sections from diabetic patients who concomitantly show increased mitochondrial fragmentation and impaired mitochondrial function [50]. Since these markers are upregulated in GRK2+/− hearts, it is tempting to suggest that these animals could present a conserved mitochondrial fusion capacity what is compatible with the improved mitochondrial respiration towards fatty acids described in murine cardiomyocytes upon GRK2 inhibition [47].

## Conclusions

**Fig. 6:**
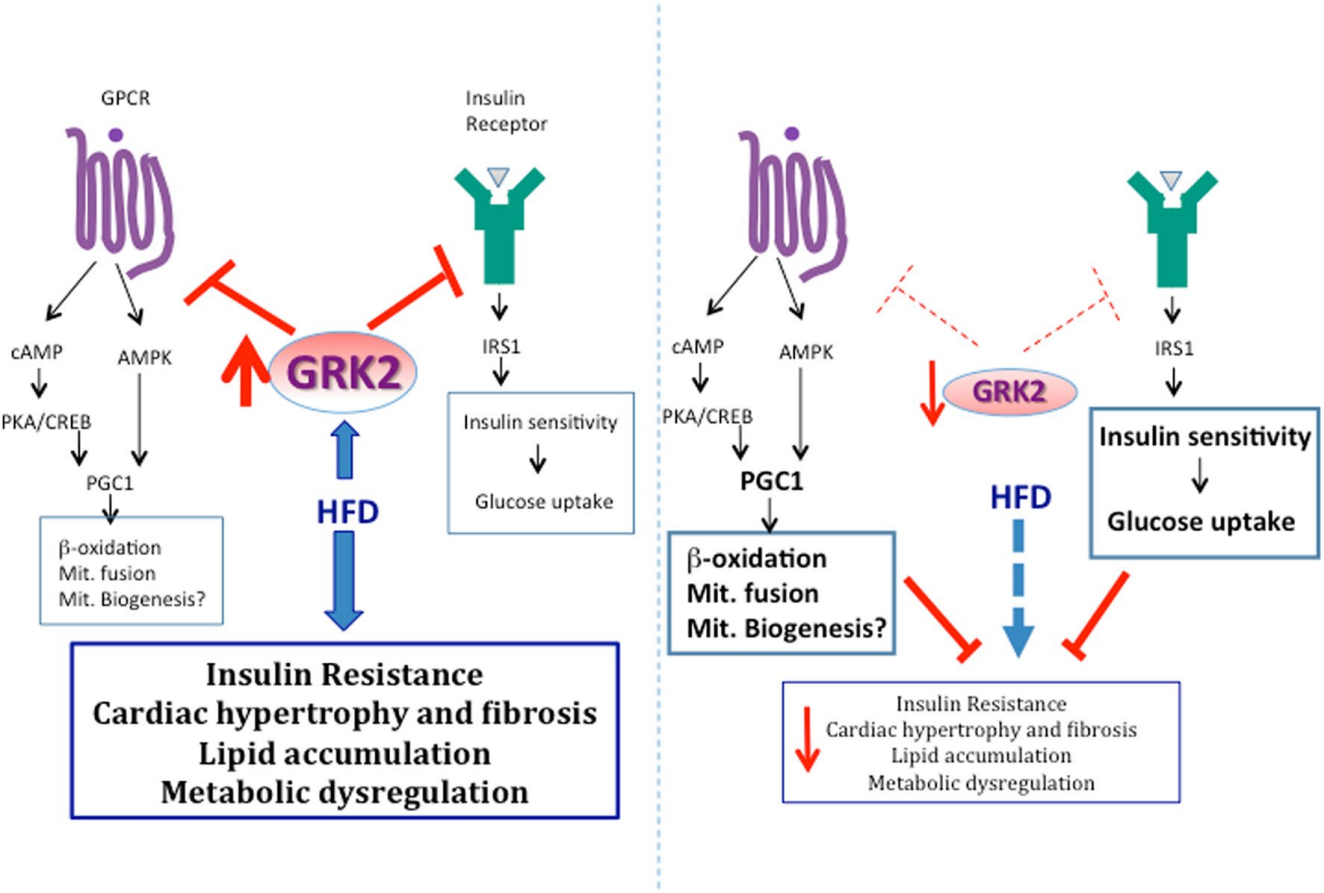
Reduced levels of GRK2 can protect cardiac tissue from hypertrophy and lipid accumulation after a long term HFD by different mechanisms

Taking these data together, we could envisage a model (Fig. 6) in which a HFD would directly or indirectly provoke an increase in cardiac GRK2 levels in parallel with (or as a cause or consequence of) heart hypertrophy, fibrosis and steatosis. This alteration in GRK2 dosage would subsequently affect GPCR signaling pathways, such as β-adrenergic, thus contributing to further disturb heart physiology and fuel cardiac metabolic remodeling by, for instance, impairing cardioprotective mechanisms downstream PKA and CREB or AMPK such as PGC1-mediated transcription of genes related to fatty acid oxidation or mitochondrial fusion. The increase in GRK2 could also contribute to further impair cardiac insulin signaling decreasing the ability of cardiac cells to uptake glucose and making them less energetically efficient. This would result in inefficient cardiac ATP production with detrimental consequences for the heart [12, 13, 47]. In such scenario, GRK2 upregulation would be a relatively early event in the development of the metabolically-induced cardiomyopathy, as has recently been suggested [47], and as is the case for other cardiac conditions in which GRK2 levels increase in the first stages of the heart disease [16].

In summary, our results point to a protective role of low levels of GRK2 upon obesity-induced cardiac remodeling and steatosis. The protection afforded by GRK2 downmodulation is apparent in the presence of concurrent relevant risk factors for insulin resistance and cardiovascular disease such as age and obesity after long-term HFD feeding. It is tempting to hypothesize that the increase in cardiac GRK2 protein levels triggered as a consequence of HFD feeding or in different pathophysiological situations would play a central role allowing progression to maladaptive tissue and metabolic cardiac remodeling due to its unique ability to simultaneously alter GPCR and insulin signaling.

## Additional file

**Additional file 1: Figure S1.** A) Weight gain induced by 30 weeks of HFD feeding in WT and GRK2+/− genotypes expressed as fold-increase over control SD-fed mice (N = 5–7). Data are mean ± SEM. ++p < 0.01; +p<0.05 referred to SD-fed mice; *p < 0.05 referred to fold increase between genotypes. B) Final body weight after 30 weeks of SD or HFD feeding in WT and GRK2+/− genotypes (N = 5–7). Statistical analysis was performed using unpaired two-tail Student’s t test. *p < 0.05, **p < 0.01. **Figure S2.** Circulating plasma NEFA concentrations in 30-week SD and HFD-fed control and GRK2+/− mice upon 16 h of fasting. Data are represented as mean ± SEM of 6–9 mice per group. Statistical significance was analyzed unpaired two-tail Student’s t test. *p < 0.05. **Figure S3.** mRNA levels of TFAM and COI genes were quantified by qPCR and normalized by a geometrical mean of HPRT and RPS29. Data are mean ± SEM (N = 5–6 per group) and the statistical analysis used was a unpaired two-tail Student’s t test. **Table S1.** Sequence of the primers used for RT-PCR analysis.

## Abbreviations

GRK2: G protein-coupled receptor kinase 2
HFD: high fat diet
GPCR: G protein-coupled receptors
NEFA: non-esterified fatty acids
PGC1: peroxisome proliferator-activated receptor gamma co-activators
PPAR: peroxisome proliferator-activated receptor
GAPDH: glyceraldehyde 3-phosphate dehydrogenase
MFN1: mitofusin-1
OPA1: optic atrophy 1
PKA: protein kinase A
CREB: cyclic AMP responsive element binding protein
AMPK: AMP-activated protein kinase
βAR: beta-adrenergic receptors
COI: cytochrome c oxidase subunit I
TFAM: mitochondrial transcription factor A
