# Supplementary material for: Obesity-induced cardiac lipid accumulation in adult mice is modulated by G protein-coupled receptor kinase 2 levels
Source: Cardiovasc Diabetol. 2016 Nov 10;15:155. doi: 10.1186/s12933-016-0474-6 (PMC5105284; doi:10.1186/s12933-016-0474-6)
Supplement: Supplementary file 1 — Additional file 1: Figure S1. A) Weight gain induced by 30 weeks of HFD feeding in WT and GRK2+/− genotypes expressed as fold-increase over control SD-fed mice (N = 5–7). Data are mean ± SEM. ++p < 0.01; +p<0.05 referred to SD-fed mice; *p < 0.05 referred to fold increase between genotypes. B) Final body weight after 30 weeks of SD or HFD feeding in WT and GRK2+/− genotypes (N = 5–7). Statistical analysis was performed using unpaired two-tail Student’s t test. *p < 0.05, **p < 0.01. Figure S2. Circulating plasma NEFA concentrations in 30-week SD and HFD-fed control and GRK2+/− mice upon 16 h of fasting. Data are represented as mean ± SEM of 6–9 mice per group. Statistical significance was analyzed unpaired two-tail Student’s t test. *p < 0.05. Figure S3. mRNA levels of TFAM and COI genes were quantified by qPCR and normalized by a geometrical mean of HPRT and RPS29. Data are mean ± SEM (N = 5–6 per group) and the statistical analysis used was a unpaired two-tail Student’s t test. Table S1. Sequence of the primers used for RT-PCR analysis. [file 12933_2016_474_MOESM1_ESM.docx]

**SUPPLEMENTARY MATERIAL** (Lucas et al)

**

**B**

| 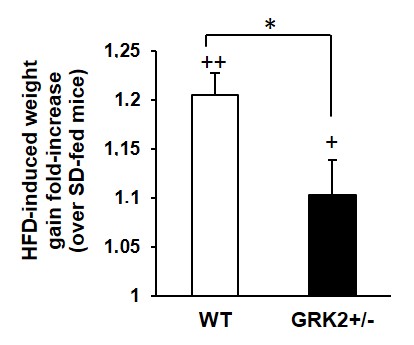  **A** |   **  **  *  **Final body weight (g)**  **SD**  **HFD** |
| --- | --- |

**Figure S1**: A) Weight gain induced by 30 weeks of HFD feeding in WT and GRK2+/- genotypes expressed as fold-increase over control SD-fed mice (N=5-7 per genotype and condition). Data are means±SEM. ++p<0.01; +p<0.05 referred to SD-fed mice; *p<0.05 referred to fold increase between genotypes. B) Final body weight after 30 weeks of SD or HFD feeding in WT and GRK2+/- genotypes (N = 5-7). Data are means±SEM. Statistical analysis was performed using unpaired two-tail Student’s t-test. *p<0.05, **p<0.01

*

**Figure S2**: Circulating plasma NEFA concentrations in 30-week SD and HFD-fed control and GRK2+/- mice upon 16 hours of fasting. Data are represented as means ± SEM of 6-9 mice per group. Statistical significance was analyzed by unpaired two-tail Student’s t-test. *p<0.05

|  **HFD**  **SD**  **A** |  **HFD**  **SD**  **B** |
| --- | --- |

**Figure S3:** mRNA levels of TFAM and COI genes were quantified by qPCR and normalized by a geometrical mean of HPRT and RPS29. Data are means ± SEM (N=5-6 per group) and the statistical analysis used was a unpaired two-tail Student’s t-test.

**Table S1.** Primers used for RT-PCR analysis.

| *acta1* |  | *TaqMan® Gene Expression Assay (Applied Biosystems):*  *Ref. Mm00808218_g1* |
| --- | --- | --- |
| *atp2a2* (serca2) |  | *TaqMan® Gene Expression Assay (Applied Biosystems):*  *Ref. Mm01201431_m1* |
| *adrbk1* | Forward  Reverse | *5´-CATGCACAATCGCTTTGTAGTC-3′*  *5´ -GGTCCGAGATTCTCACATGG-3′* |
| *bnp* | Forward  Reverse | *5´-GTCAGTCGTTTGGGCTGTAAC-3′*  *5´-AGACCCAGGCAGAGTCAGAA-3′* |
| *mt-coI* | Forward  Reverse | *5´-TCTCCTTCTCCTAGCATCATCA-3′*  *5´-CCGGCTAGAGGTGGGTAGA-3′* |
| *hprt1* | Forward  Reverse | *5´ -TCCTCCTCAGACCGCTTTT-3´*  *5´ -CCTGGTTCATCATCGCTAATC-3´* |
| *ppara* | Forward  Reverse | *5´ -CACGCATGTGAAGGCTGTAA-3´*  *5´ -CAGCTCCGATCACACTTGTC-3´* |
| *ppargc1a* | Forward  Reverse | *5´ -CCCTTCTTTGCCATTGAATC -3′*  *5´ -AATGTTAGGAAAGTTTAGCATCTGG-3′* |
| *ppargc1b* | Forward  Reverse | *5´ -GGCGCGCTGCTGGAT-3´*  *5´ -CTCCAGAGTCCCCACCCTG-3´* |
| *rps29* | Forward  Reverse | *5´ -CTGAACATGTGCCGCCAGT-3′*  *5´ -TCAAGGTCGCTTAGTCCAACTTAAT-3′* |
| *tfam* | Forward  Reverse | *5´ -ATTGACCATGTGCTTCAGAGC-3′*  *5´ -GCATCCCTGTATCGCTGTAGT-3′* |
